# 7-(*tert*-Butyl­diphenyl­sil­yloxy)-2,2-dimethyl-1-benzofuran-3(2*H*)-one

**DOI:** 10.1107/S1600536810054462

**Published:** 2011-01-12

**Authors:** Cristian O. Salas, Ricardo A. Tapia, Alejandro Macías

**Affiliations:** a Departamento de Química Orgánica, Facultad de Química, Pontificia Universidad Católica de Chile, 702843 Santiago de Chile, Chile; b Departamento de Química Inorgánica, Universidad de Santiago de Compostela, 15782 Santiago de Compostela, Spain

## Abstract

The title compound, C_26_H_28_O_3_Si, is an allylic oxidation product of the *tert*-but­yl(2,2-dimethyl-2,3-dihydro­benzo­furan-7-yl­oxy)diphenyl­silane with *N*-bromo­succinimide and 2,2′-azobis-isobutyronitrile. The nine-atom bicyclic system is almost planar, with an r.m.s deviation of 0.0123 (2) Å and a maximum deviation of 0.031 (2) Å for the O atom. In the crystal, the mol­ecules pile up along the *b* axis but the strongest inter­molecular contacts are the π–π stacking inter­actions between the benzene rings along the *c* axis [centroid–centroid distance = 3.655 (3) Å].

## Related literature

Benzofuran­ones are precursors of a wide range of natural and synthetic products. For a related transformation of benzo­furan­ones in aurones, see: Schoepfer *et al.* (2002 ▶); Löser *et al.* (2004 ▶); in spiro­annulated and aromatic spiro­ketal compounds, see: Braun *et al.* (2008 ▶); Zhou *et al.* (2008 ▶); in benzofurane derivatives, see: Venkatesan *et al.*(2010 ▶); and in pyran­o­benzofuranes, see: Foroumadi *et al.* (2009 ▶).
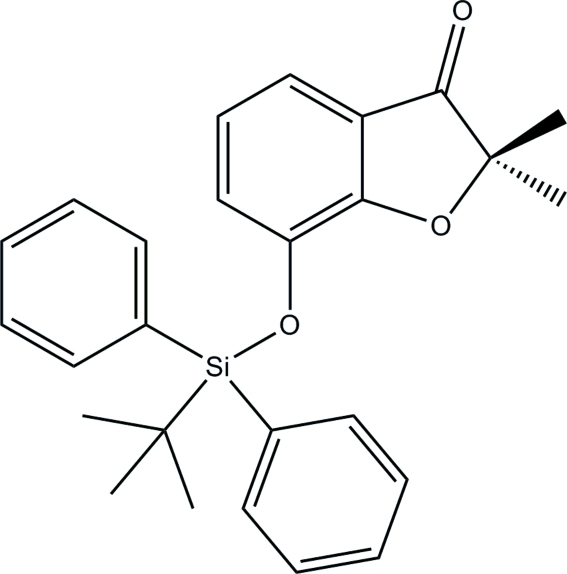


## Experimental

### 

#### Crystal data

C_26_H_28_O_3_Si
*M* *_r_* = 416.57
Triclinic, 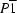

*a* = 9.8210 (18) Å
*b* = 11.081 (2) Å
*c* = 12.025 (2) Å
α = 98.803 (2)°
β = 112.151 (2)°
γ = 101.791 (2)°
*V* = 1147.7 (4) Å^3^
*Z* = 2
Mo *K*α radiation
μ = 0.13 mm^−1^
*T* = 100 K
0.49 × 0.43 × 0.10 mm

#### Data collection

Bruker SMART 1000 CCD diffractometer
Absorption correction: multi-scan (*SADABS*; Bruker, 2001 ▶) *T*_min_ = 0.941, *T*_max_ = 0.988
14369 measured reflections
4197 independent reflections
3325 reflections with *I* > 2σ(*I*)
*R*_int_ = 0.032

#### Refinement

*R*[*F* ^2^ > 2σ(*F* ^2^)] = 0.044
*wR*(*F* ^2^) = 0.111
*S* = 1.05
4197 reflections
276 parameters
H-atom parameters constrained
Δρ_max_ = 0.83 e Å^−3^
Δρ_min_ = −0.29 e Å^−3^

### 

Data collection: *SMART* (Bruker, 2007 ▶); cell refinement: *SAINT* (Bruker, 2007 ▶); data reduction: *SAINT*; program(s) used to solve structure: *SIR97* (Altomare *et al.*, 1999 ▶); program(s) used to refine structure: *SHELXL97* (Sheldrick, 2008 ▶); molecular graphics: *ORTEP-3 for Windows* (Farrugia, 1997 ▶); software used to prepare material for publication: *WinGX* (Farrugia, 1999 ▶).

## Supplementary Material

Crystal structure: contains datablocks I, global. DOI: 10.1107/S1600536810054462/si2320sup1.cif

Structure factors: contains datablocks I. DOI: 10.1107/S1600536810054462/si2320Isup2.hkl

Additional supplementary materials:  crystallographic information; 3D view; checkCIF report

## Figures and Tables

**Fig. 1.**
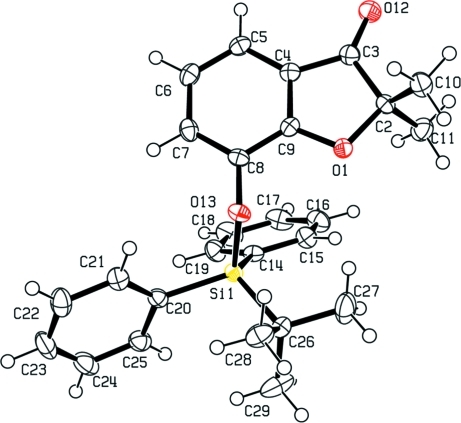
The structure of the title compound, with displacement ellipsoids drawn at the 50% probability level and H atoms with arbitrary radius.

**Fig. 2.**
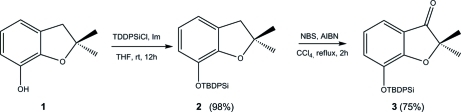
Chemical reactions scheme for obtain molecule 3.

## supplementary crystallographic information

### Comment

Benzofuranones are very important compounds because of their use in a wide range of natural and synthetic products with relevant properties such as spiroannulated benzofuranones (Braun *et al.*, 2008), aromatics spiroketals compounds (Zhou *et al.*, 2008), aurones (Schoepfer *et al*, 2002; Löser *et al.*, 2004), pyranobenzofuranes (Foroumadi *et al.*, 2009) and some benzofuranes derivatives (Venkatesan *et al.*, 2010). The benzofuranone 3 is the product of the allylic oxidation of the *tert*-butyl-(2,2-dimethyl-2,3-dihydrobenzofuran-7-yloxy)diphenylsilane with *N*-bromosuccinimide (NBS) and 2,2'-azobis-isobutyronitrile (AIBN) (Fig. 2). The molecular structure of the title compound is represented in Fig. 1. Bond lengths and angles are within the expected values and confirm the bond orders giving in the Scheme. The 9-atom bicyclic system is, as expected, planar, with r.m.s deviation = 0.0123 (2) Å and a maximum deviation of 0.031 (2) Å. The molecules pile up along the *b* axis but the strongest intermolecular contacts are the π–π stacking interactions between the benzo rings along the *c* axis [centroid–centroid distances = 3.655 (5) Å].

### Experimental

*tert*-Butyl-(2,2-dimethyl-2,3-dihydrobenzofuran-7-yloxy)diphenylsilane (2)

*tert*-Butyldiphenylsilyl chloride (1.0 g, 3.64 mmol) and imidazole (1.52 g, 22.35 mmol) were added to a solution of 2,2-dimethyl-2,3-dihydrobenzofuran-7-ol (1) (0.59 g, 3.62 mmol) in dry THF (50 ml) and the mixture was stirred at room temperature for 12 h. under an nitrogen atmosphere. Petroleum ether (100 ml) was added and the solid was filtered off and the solvents were removed *in vacuo* to give an oil residue, which was purified by flash column chromatography (CH_2_Cl_2_/ petroleum ether, 9:1) to give *tert*-butyl-(2,2-dimethyl-2,3-dihydrobenzofuran-7-yloxy)diphenylsilane (2) (1.43 g, 98%) as a colorless oil.

7-(*tert*-Butyldiphenylsilyloxy)-2,2-dimethylbenzofuran-3(2*H*)-one (3).

NBS (1.54 g, 8.70 mmol) and AIBN (25 mg) were added to a solution of *tert*-butyl(2,2-dimethyl-2,3-dihydrobenzofuran-7-yloxy)diphenylsilane (2) (1.0 g, 2.39 mmol) in dry CCl_4_ (150 ml) and the resulting suspension was stirred at reflux for 2 h. The mixture was cooled and filtered. The filtrate was evaporated to dryness *in vacuo* to give a residue, which was purified by flash column chromatography (CH_2_Cl_2_) to give 7-(*tert*-butyldiphenylsilyloxy)-2,2-dimethylbenzofuran-3(2*H*)-one (3) (0.75 g, 75%) as a white solid. mp: 354.5–355.5 K. Crystals were grown by slow evaporation from CH_2_Cl_2_.

### Refinement

H atoms were placed in idealized positions with C—H distances 0.95 – 0.98 Å and thereafter treated as riding. A torsional parameter was refined for each methyl group. *U*iso for H were assigned as 1.2 times *U*eq of the attached C atom (1.5 for the methyl groups).

### Crystal data

**Table:**

C_26_H_28_O_3_Si	*Z* = 2
*M**_r_* = 416.57	*F*(000) = 444
Triclinic, *P*1	*D*_x_ = 1.205 Mg m^−^^3^
*a* = 9.8210 (18) Å	Mo *K*α radiation, λ = 0.71073 Å
*b* = 11.081 (2) Å	Cell parameters from 3414 reflections
*c* = 12.025 (2) Å	θ = 2.3–27.3°
α = 98.803 (2)°	µ = 0.13 mm^−^^1^
β = 112.151 (2)°	*T* = 100 K
γ = 101.791 (2)°	Prism, colourless
*V* = 1147.7 (4) Å^3^	0.49 × 0.43 × 0.10 mm

### Data collection

**Table:**

Bruker SMART 1000 CCD diffractometer	4197 independent reflections
Radiation source: fine-focus sealed tube	3325 reflections with *I* > 2σ(*I*)
graphite	*R*_int_ = 0.032
ω scans	θ_max_ = 25.4°, θ_min_ = 1.9°
Absorption correction: multi-scan (*SADABS*; Bruker, 2001)	*h* = −11→10
*T*_min_ = 0.941, *T*_max_ = 0.988	*k* = −13→13
14369 measured reflections	*l* = 0→14

### Refinement

**Table:**

Refinement on *F*^2^	Primary atom site location: structure-invariant direct methods
Least-squares matrix: full	Secondary atom site location: difference Fourier map
*R*[*F*^2^ > 2σ(*F*^2^)] = 0.044	Hydrogen site location: inferred from neighbouring sites
*wR*(*F*^2^) = 0.111	H-atom parameters constrained
*S* = 1.05	*w* = 1/[σ^2^(*F*_o_^2^) + (0.0422*P*)^2^ + 0.8404*P*] where *P* = (*F*_o_^2^ + 2*F*_c_^2^)/3
4197 reflections	(Δ/σ)_max_ < 0.001
276 parameters	Δρ_max_ = 0.83 e Å^−^^3^
0 restraints	Δρ_min_ = −0.29 e Å^−^^3^

### Special details

**Table:**

Experimental. *tert*-Butyl-(2,2-dimethyl-2,3-dihydrobenzofuran-7-yloxy)diphenylsilane (2)^1^H RMN (CDCl_3_, 200 MHz) d 1.18 (s, 9H, 3xCH_3_); 1.34 (s, 6H, 2xCH_3_), 2.96 (s, 2H, H3); 6.53–6.56 (m, 2H, H5, H4); 6.70 (m, 1H, H6); 7.35–7.44 (m, 6H, H—Ar); 7.38–7.83 (m, 4H, H—Ar). ^13^C RMN (CDCl_3_, 50 MHz) d 19.7 (C(CH_3_)_3_); 26.8 (3xCH_3_); 28.1 (2xCH_3_); 43.4 (C3); 86.4 (C2); 117.9 (C6); 119.6 (C5); 119.8 (C4); 127.5 (Ar); 127.8 (C3a); 129.6 (Ar); 133.9 (Ar); 135.7 (Ar); 139.9 (C7); 149.4 (C7a).7-(*tert*-Butyldiphenylsilyloxy)-2,2-dimethylbenzofuran-3(2*H*)-one (3).IR (NaCl, cm^-1^): 1714 (CO). ^1^H-RMN (CDCl_3_, 200 MHz) d 1.18 (s, 9H, 3xCH_3_); 1.30 (s, 6H, 2xCH_3_); 6.74 (t, 1H, *J* =7.7 Hz, H5,); 6.97 (dd, 1H, *J* =1.1, *J* =7.8 Hz, H6); 7.21 (dd, 1H, *J* =1,1, *J* =7,6 Hz, H4); 7.32–7.48 (m, 6H, H—Ar); 7.72–7.77 (m, 4H, H—Ar). ^13^C-RMN (CDCl_3_, 50 MHz) d 19.7 (C(CH_3_)_3_); 22.8 (3xCH_3_); 26.6 (2xCH_3_); 87.9 (C2); 117.0 (C5); 121.0 (C7); 121.8 (C4); 127.7 (Ar); 130.0 (Ar); 133.0 (C6); 135.5 (Ar); 142.4 (C3a); 162.4 (C7a); 204.8 (C3). MS (CI) *m*/*z* 417 [(*M*^+^, 74]; 359 (66); 339 (100).
Geometry. All s.u.'s (except the s.u. in the dihedral angle between two l.s. planes) are estimated using the full covariance matrix. The cell s.u.'s are taken into account individually in the estimation of s.u.'s in distances, angles and torsion angles; correlations between s.u.'s in cell parameters are only used when they are defined by crystal symmetry. An approximate (isotropic) treatment of cell s.u.'s is used for estimating s.u.'s involving l.s. planes.
Refinement. Refinement of *F*^2^ against ALL reflections. The weighted *R*-factor *wR* and goodness of fit *S* are based on *F*^2^, conventional *R*-factors *R* are based on *F*, with *F* set to zero for negative *F*^2^. The threshold expression of *F*^2^ > 2σ(*F*^2^) is used only for calculating *R*-factors(gt) *etc*. and is not relevant to the choice of reflections for refinement. *R*-factors based on *F*^2^ are statistically about twice as large as those based on *F*, and *R*- factors based on ALL data will be even larger.

### Fractional atomic coordinates and isotropic or equivalent isotropic displacement parameters (Å^2^)

**Table:**

	*x*	*y*	*z*	*U*_iso_*/*U*_eq_	
Si1	0.05281 (6)	0.33072 (5)	0.76624 (5)	0.01833 (15)	
O1	0.32466 (15)	0.13402 (12)	0.72764 (12)	0.0227 (3)	
C2	0.4315 (2)	0.05650 (19)	0.75715 (18)	0.0223 (4)	
C3	0.3300 (2)	−0.07888 (19)	0.73025 (18)	0.0227 (4)	
C4	0.1727 (2)	−0.07133 (18)	0.68675 (17)	0.0202 (4)	
C5	0.0325 (2)	−0.16301 (19)	0.64733 (18)	0.0232 (4)	
H5	0.0270	−0.2501	0.6447	0.028*	
C6	−0.0980 (2)	−0.1229 (2)	0.61233 (18)	0.0252 (5)	
H6	−0.1955	−0.1833	0.5839	0.030*	
C7	−0.0885 (2)	0.0057 (2)	0.61825 (18)	0.0236 (4)	
H7	−0.1800	0.0311	0.5953	0.028*	
C8	0.0501 (2)	0.09704 (18)	0.65653 (17)	0.0193 (4)	
C9	0.1812 (2)	0.05545 (18)	0.68970 (16)	0.0188 (4)	
C10	0.5136 (2)	0.0626 (2)	0.6729 (2)	0.0296 (5)	
H10A	0.4379	0.0327	0.5860	0.044*	
H10B	0.5824	0.0083	0.6894	0.044*	
H10C	0.5734	0.1508	0.6886	0.044*	
C11	0.5402 (2)	0.1070 (2)	0.8933 (2)	0.0316 (5)	
H11A	0.5988	0.1955	0.9086	0.047*	
H11B	0.6109	0.0548	0.9159	0.047*	
H11C	0.4813	0.1033	0.9434	0.047*	
O12	0.37919 (16)	−0.17058 (14)	0.74359 (14)	0.0320 (4)	
O13	0.06209 (15)	0.22261 (12)	0.65909 (12)	0.0217 (3)	
C14	0.0978 (2)	0.27196 (18)	0.90971 (18)	0.0212 (4)	
C15	0.2433 (2)	0.2591 (2)	0.97519 (19)	0.0270 (5)	
H15	0.3220	0.2867	0.9493	0.032*	
C16	0.2760 (3)	0.2073 (2)	1.0766 (2)	0.0323 (5)	
H16	0.3764	0.2007	1.1200	0.039*	
C17	0.1623 (3)	0.1654 (2)	1.1145 (2)	0.0358 (6)	
H17	0.1840	0.1291	1.1837	0.043*	
C18	0.0170 (3)	0.1763 (2)	1.0515 (2)	0.0371 (6)	
H18	−0.0613	0.1476	1.0776	0.045*	
C19	−0.0147 (2)	0.2288 (2)	0.9508 (2)	0.0285 (5)	
H19	−0.1151	0.2358	0.9084	0.034*	
C20	−0.1450 (2)	0.34886 (18)	0.70512 (18)	0.0215 (4)	
C21	−0.2426 (2)	0.3037 (2)	0.57890 (19)	0.0253 (5)	
H21	−0.2115	0.2558	0.5252	0.030*	
C22	−0.3847 (2)	0.3276 (2)	0.5300 (2)	0.0337 (5)	
H22	−0.4498	0.2958	0.4438	0.040*	
C23	−0.4303 (3)	0.3977 (2)	0.6075 (2)	0.0379 (6)	
H23	−0.5273	0.4139	0.5748	0.045*	
C24	−0.3351 (3)	0.4442 (2)	0.7324 (2)	0.0365 (6)	
H24	−0.3665	0.4928	0.7854	0.044*	
C25	−0.1943 (2)	0.4205 (2)	0.7807 (2)	0.0281 (5)	
H25	−0.1298	0.4534	0.8668	0.034*	
C26	0.1895 (2)	0.48211 (19)	0.77629 (18)	0.0220 (4)	
C27	0.3543 (3)	0.4743 (2)	0.8183 (3)	0.0462 (7)	
H27A	0.3558	0.3973	0.7669	0.069*	
H27B	0.3951	0.4711	0.9054	0.069*	
H27C	0.4179	0.5495	0.8094	0.069*	
C28	0.1375 (3)	0.5049 (2)	0.6466 (2)	0.0318 (5)	
H28A	0.2037	0.5858	0.6497	0.048*	
H28B	0.0312	0.5086	0.6170	0.048*	
H28C	0.1442	0.4351	0.5899	0.048*	
C29	0.1862 (3)	0.5957 (2)	0.8649 (2)	0.0428 (6)	
H29A	0.2554	0.6739	0.8663	0.064*	
H29B	0.2195	0.5823	0.9486	0.064*	
H29C	0.0816	0.6034	0.8363	0.064*	

### Atomic displacement parameters (Å^2^)

**Table:**

	*U*^11^	*U*^22^	*U*^33^	*U*^12^	*U*^13^	*U*^23^
Si1	0.0189 (3)	0.0199 (3)	0.0175 (3)	0.0066 (2)	0.0087 (2)	0.0046 (2)
O1	0.0195 (7)	0.0194 (7)	0.0283 (8)	0.0056 (6)	0.0088 (6)	0.0070 (6)
C2	0.0201 (10)	0.0210 (10)	0.0256 (11)	0.0085 (8)	0.0076 (8)	0.0069 (8)
C3	0.0243 (10)	0.0234 (11)	0.0208 (10)	0.0083 (9)	0.0085 (8)	0.0074 (8)
C4	0.0240 (10)	0.0208 (10)	0.0156 (9)	0.0069 (8)	0.0079 (8)	0.0050 (8)
C5	0.0268 (11)	0.0190 (10)	0.0223 (10)	0.0033 (8)	0.0104 (9)	0.0050 (8)
C6	0.0222 (10)	0.0266 (11)	0.0236 (11)	0.0009 (9)	0.0100 (9)	0.0052 (9)
C7	0.0205 (10)	0.0326 (12)	0.0201 (10)	0.0096 (9)	0.0101 (8)	0.0069 (9)
C8	0.0259 (10)	0.0199 (10)	0.0158 (9)	0.0094 (8)	0.0112 (8)	0.0047 (8)
C9	0.0204 (10)	0.0206 (10)	0.0143 (9)	0.0031 (8)	0.0080 (8)	0.0034 (8)
C10	0.0266 (11)	0.0322 (12)	0.0336 (12)	0.0105 (9)	0.0142 (10)	0.0112 (10)
C11	0.0284 (12)	0.0297 (12)	0.0289 (12)	0.0059 (9)	0.0057 (10)	0.0058 (9)
O12	0.0287 (8)	0.0236 (8)	0.0416 (9)	0.0108 (7)	0.0098 (7)	0.0110 (7)
O13	0.0280 (8)	0.0216 (7)	0.0207 (7)	0.0119 (6)	0.0128 (6)	0.0069 (6)
C14	0.0268 (11)	0.0175 (10)	0.0200 (10)	0.0074 (8)	0.0104 (8)	0.0037 (8)
C15	0.0280 (11)	0.0293 (12)	0.0264 (11)	0.0100 (9)	0.0122 (9)	0.0095 (9)
C16	0.0369 (13)	0.0359 (13)	0.0252 (11)	0.0173 (10)	0.0097 (10)	0.0105 (10)
C17	0.0558 (16)	0.0353 (13)	0.0273 (12)	0.0228 (12)	0.0210 (11)	0.0163 (10)
C18	0.0502 (15)	0.0418 (14)	0.0389 (13)	0.0194 (12)	0.0320 (12)	0.0210 (11)
C19	0.0288 (12)	0.0326 (12)	0.0320 (12)	0.0124 (10)	0.0176 (10)	0.0124 (10)
C20	0.0228 (10)	0.0199 (10)	0.0260 (11)	0.0077 (8)	0.0126 (9)	0.0096 (8)
C21	0.0257 (11)	0.0277 (11)	0.0259 (11)	0.0085 (9)	0.0122 (9)	0.0117 (9)
C22	0.0250 (11)	0.0424 (14)	0.0337 (13)	0.0103 (10)	0.0083 (10)	0.0196 (11)
C23	0.0256 (12)	0.0436 (14)	0.0563 (16)	0.0199 (11)	0.0193 (11)	0.0265 (12)
C24	0.0360 (13)	0.0348 (13)	0.0538 (16)	0.0188 (11)	0.0286 (12)	0.0160 (12)
C25	0.0295 (12)	0.0258 (11)	0.0336 (12)	0.0089 (9)	0.0175 (10)	0.0079 (9)
C26	0.0208 (10)	0.0221 (10)	0.0208 (10)	0.0026 (8)	0.0087 (8)	0.0043 (8)
C27	0.0239 (12)	0.0448 (15)	0.0687 (18)	0.0044 (11)	0.0147 (12)	0.0314 (14)
C28	0.0398 (13)	0.0257 (12)	0.0271 (11)	0.0042 (10)	0.0131 (10)	0.0091 (9)
C29	0.0602 (17)	0.0235 (12)	0.0409 (14)	−0.0067 (11)	0.0316 (13)	−0.0038 (10)

### Geometric parameters (Å, °)

**Table:**

Si1—O13	1.6627 (14)	C16—C17	1.381 (3)
Si1—C14	1.866 (2)	C16—H16	0.9500
Si1—C20	1.866 (2)	C17—C18	1.381 (3)
Si1—C26	1.878 (2)	C17—H17	0.9500
O1—C9	1.358 (2)	C18—C19	1.382 (3)
O1—C2	1.465 (2)	C18—H18	0.9500
C2—C11	1.513 (3)	C19—H19	0.9500
C2—C10	1.515 (3)	C20—C21	1.396 (3)
C2—C3	1.532 (3)	C20—C25	1.400 (3)
C3—O12	1.218 (2)	C21—C22	1.394 (3)
C3—C4	1.457 (3)	C21—H21	0.9500
C4—C9	1.384 (3)	C22—C23	1.382 (3)
C4—C5	1.394 (3)	C22—H22	0.9500
C5—C6	1.379 (3)	C23—C24	1.379 (3)
C5—H5	0.9500	C23—H23	0.9500
C6—C7	1.398 (3)	C24—C25	1.381 (3)
C6—H6	0.9500	C24—H24	0.9500
C7—C8	1.383 (3)	C25—H25	0.9500
C7—H7	0.9500	C26—C28	1.526 (3)
C8—O13	1.367 (2)	C26—C27	1.529 (3)
C8—C9	1.393 (3)	C26—C29	1.534 (3)
C10—H10A	0.9800	C27—H27A	0.9800
C10—H10B	0.9800	C27—H27B	0.9800
C10—H10C	0.9800	C27—H27C	0.9800
C11—H11A	0.9800	C28—H28A	0.9800
C11—H11B	0.9800	C28—H28B	0.9800
C11—H11C	0.9800	C28—H28C	0.9800
C14—C15	1.397 (3)	C29—H29A	0.9800
C14—C19	1.400 (3)	C29—H29B	0.9800
C15—C16	1.383 (3)	C29—H29C	0.9800
C15—H15	0.9500		
			
O13—Si1—C14	107.62 (8)	C17—C16—H16	120.1
O13—Si1—C20	108.10 (8)	C15—C16—H16	120.1
C14—Si1—C20	111.46 (9)	C18—C17—C16	119.8 (2)
O13—Si1—C26	103.79 (8)	C18—C17—H17	120.1
C14—Si1—C26	116.95 (9)	C16—C17—H17	120.1
C20—Si1—C26	108.36 (9)	C17—C18—C19	120.2 (2)
C9—O1—C2	107.46 (14)	C17—C18—H18	119.9
O1—C2—C11	107.94 (16)	C19—C18—H18	119.9
O1—C2—C10	108.69 (16)	C18—C19—C14	121.5 (2)
C11—C2—C10	112.76 (17)	C18—C19—H19	119.3
O1—C2—C3	104.86 (15)	C14—C19—H19	119.3
C11—C2—C3	111.31 (17)	C21—C20—C25	117.43 (18)
C10—C2—C3	110.89 (17)	C21—C20—Si1	120.75 (15)
O12—C3—C4	129.89 (19)	C25—C20—Si1	121.41 (16)
O12—C3—C2	123.83 (18)	C22—C21—C20	121.4 (2)
C4—C3—C2	106.28 (16)	C22—C21—H21	119.3
C9—C4—C5	121.40 (18)	C20—C21—H21	119.3
C9—C4—C3	106.16 (17)	C23—C22—C21	119.6 (2)
C5—C4—C3	132.44 (18)	C23—C22—H22	120.2
C6—C5—C4	117.65 (19)	C21—C22—H22	120.2
C6—C5—H5	121.2	C24—C23—C22	120.0 (2)
C4—C5—H5	121.2	C24—C23—H23	120.0
C5—C6—C7	120.71 (19)	C22—C23—H23	120.0
C5—C6—H6	119.6	C23—C24—C25	120.3 (2)
C7—C6—H6	119.6	C23—C24—H24	119.8
C8—C7—C6	121.96 (18)	C25—C24—H24	119.8
C8—C7—H7	119.0	C24—C25—C20	121.3 (2)
C6—C7—H7	119.0	C24—C25—H25	119.4
O13—C8—C7	123.14 (17)	C20—C25—H25	119.4
O13—C8—C9	119.85 (17)	C28—C26—C27	107.98 (18)
C7—C8—C9	116.99 (18)	C28—C26—C29	108.62 (18)
O1—C9—C4	115.22 (17)	C27—C26—C29	109.12 (19)
O1—C9—C8	123.50 (17)	C28—C26—Si1	107.54 (14)
C4—C9—C8	121.27 (17)	C27—C26—Si1	112.54 (15)
C2—C10—H10A	109.5	C29—C26—Si1	110.90 (14)
C2—C10—H10B	109.5	C26—C27—H27A	109.5
H10A—C10—H10B	109.5	C26—C27—H27B	109.5
C2—C10—H10C	109.5	H27A—C27—H27B	109.5
H10A—C10—H10C	109.5	C26—C27—H27C	109.5
H10B—C10—H10C	109.5	H27A—C27—H27C	109.5
C2—C11—H11A	109.5	H27B—C27—H27C	109.5
C2—C11—H11B	109.5	C26—C28—H28A	109.5
H11A—C11—H11B	109.5	C26—C28—H28B	109.5
C2—C11—H11C	109.5	H28A—C28—H28B	109.5
H11A—C11—H11C	109.5	C26—C28—H28C	109.5
H11B—C11—H11C	109.5	H28A—C28—H28C	109.5
C8—O13—Si1	126.65 (12)	H28B—C28—H28C	109.5
C15—C14—C19	116.86 (18)	C26—C29—H29A	109.5
C15—C14—Si1	120.96 (15)	C26—C29—H29B	109.5
C19—C14—Si1	121.97 (15)	H29A—C29—H29B	109.5
C16—C15—C14	121.9 (2)	C26—C29—H29C	109.5
C16—C15—H15	119.0	H29A—C29—H29C	109.5
C14—C15—H15	119.0	H29B—C29—H29C	109.5
C17—C16—C15	119.7 (2)		
			
C9—O1—C2—C11	−118.39 (17)	C26—Si1—C14—C15	−50.63 (19)
C9—O1—C2—C10	119.01 (17)	O13—Si1—C14—C19	−108.91 (17)
C9—O1—C2—C3	0.37 (19)	C20—Si1—C14—C19	9.4 (2)
O1—C2—C3—O12	179.64 (18)	C26—Si1—C14—C19	134.85 (17)
C11—C2—C3—O12	−63.9 (3)	C19—C14—C15—C16	−0.6 (3)
C10—C2—C3—O12	62.5 (3)	Si1—C14—C15—C16	−175.34 (17)
O1—C2—C3—C4	0.30 (19)	C14—C15—C16—C17	0.8 (3)
C11—C2—C3—C4	116.76 (18)	C15—C16—C17—C18	−0.5 (3)
C10—C2—C3—C4	−116.84 (18)	C16—C17—C18—C19	0.1 (4)
O12—C3—C4—C9	179.9 (2)	C17—C18—C19—C14	0.1 (4)
C2—C3—C4—C9	−0.8 (2)	C15—C14—C19—C18	0.1 (3)
O12—C3—C4—C5	−0.2 (4)	Si1—C14—C19—C18	174.87 (17)
C2—C3—C4—C5	179.0 (2)	O13—Si1—C20—C21	−16.23 (18)
C9—C4—C5—C6	−0.6 (3)	C14—Si1—C20—C21	−134.30 (16)
C3—C4—C5—C6	179.51 (19)	C26—Si1—C20—C21	95.65 (17)
C4—C5—C6—C7	−0.9 (3)	O13—Si1—C20—C25	171.27 (15)
C5—C6—C7—C8	1.3 (3)	C14—Si1—C20—C25	53.20 (19)
C6—C7—C8—O13	177.67 (17)	C26—Si1—C20—C25	−76.85 (18)
C6—C7—C8—C9	−0.2 (3)	C25—C20—C21—C22	−0.8 (3)
C2—O1—C9—C4	−1.0 (2)	Si1—C20—C21—C22	−173.60 (16)
C2—O1—C9—C8	178.51 (17)	C20—C21—C22—C23	0.3 (3)
C5—C4—C9—O1	−178.72 (16)	C21—C22—C23—C24	0.3 (3)
C3—C4—C9—O1	1.2 (2)	C22—C23—C24—C25	−0.3 (3)
C5—C4—C9—C8	1.8 (3)	C23—C24—C25—C20	−0.2 (3)
C3—C4—C9—C8	−178.34 (17)	C21—C20—C25—C24	0.8 (3)
O13—C8—C9—O1	1.3 (3)	Si1—C20—C25—C24	173.55 (16)
C7—C8—C9—O1	179.22 (17)	O13—Si1—C26—C28	56.95 (15)
O13—C8—C9—C4	−179.26 (16)	C14—Si1—C26—C28	175.28 (13)
C7—C8—C9—C4	−1.3 (3)	C20—Si1—C26—C28	−57.78 (16)
C7—C8—O13—Si1	78.3 (2)	O13—Si1—C26—C27	−61.85 (17)
C9—C8—O13—Si1	−103.83 (18)	C14—Si1—C26—C27	56.47 (19)
C14—Si1—O13—C8	21.23 (17)	C20—Si1—C26—C27	−176.58 (16)
C20—Si1—O13—C8	−99.27 (16)	O13—Si1—C26—C29	175.59 (15)
C26—Si1—O13—C8	145.81 (15)	C14—Si1—C26—C29	−66.09 (18)
O13—Si1—C14—C15	65.61 (18)	C20—Si1—C26—C29	60.85 (17)
C20—Si1—C14—C15	−176.04 (16)		
